# Combinatorial Approach to Improve Cancer Immunotherapy: Rational Drug Design Strategy to Simultaneously Hit Multiple Targets to Kill Tumor Cells and to Activate the Immune System

**DOI:** 10.1155/2019/5245034

**Published:** 2019-02-03

**Authors:** Shweta Joshi, Donald L. Durden

**Affiliations:** ^1^Division of Pediatric Hematology-Oncology, Department of Pediatrics, Moores Cancer Center, University of California, San Diego, CA, USA; ^2^SignalRx Pharmaceuticals, Inc., San Diego, CA, USA

## Abstract

Cancer immunotherapy, including immune checkpoint blockade and adoptive CAR T-cell therapy, has clearly established itself as an important modality to treat melanoma and other malignancies. Despite the tremendous clinical success of immunotherapy over other cancer treatments, this approach has shown substantial benefit to only some of the patients while the rest of the patients have not responded due to immune evasion. In recent years, a combination of cancer immunotherapy together with existing anticancer treatments has gained significant attention and has been extensively investigated in preclinical or clinical studies. In this review, we discuss the therapeutic potential of novel regimens combining immune checkpoint inhibitors with therapeutic interventions that (1) increase tumor immunogenicity such as chemotherapy, radiotherapy, and epigenetic therapy; (2) reverse tumor immunosuppression such as TAMs, MDSCs, and Tregs targeted therapy; and (3) reduce tumor burden and increase the immune effector response with rationally designed dual or triple inhibitory chemotypes.

## 1. Introduction

The ultimate aim of immunotherapy is to boost the body's immune system to destroy tumor cells and to provide a durable antitumor immune response. The strategy of using monoclonal antibodies against two distinct inhibitory receptors on T-cells, PD1, and CTLA-4 is a major breakthrough in the field of cancer immunotherapy. The efficacy of this strategy was first established in patients with metastatic melanoma based on the antitumor immune response and increased overall survival rates of patients treated with ipilimumab, a monoclonal antibody targeting human CTLA-4 [1]. The remarkable antitumor activity of PD-1/PDL-1 inhibition in melanoma, renal cell carcinoma, and NSCLC lead to regulatory approval of increasing list of anti-PD1/PDL1 antibodies in hematological malignancies and various other solid cancers [2, 3]. Nevertheless, the efficacy of PD-1/PD-L1 pathway inhibition as a monotherapy has provided benefit to only some of the patients while a significant fraction does not respond to this therapy.

Analysis of clinical trial data suggests three types of patients: (a) those that do not respond (innate resistance); (b) those that respond initially but fail to respond in later stages (acquired resistance); and (c) those that respond initially and continue to respond [4, 5]. Extensive research has been performed in the past few years to understand the mechanisms that regulate immune response to cancer, but obstacles still exist in the field of cancer immunotherapy. Mechanisms of innate and acquired resistance to PD1/PDL1 blockade have been excellently reviewed before [6, 7]. In order to generate an efficient antitumor immune response, activation and proliferation of antigen experienced T-cells are required; due to inadequate generation and function of tumor-reactive CD8 T-cells, patients do not respond to this therapy [8]. Scarcity of suitable neoantigens and impaired processing and presentation of neoantigens are other reasons that lead to ineffective activation of tumor-reactive T-cells [5]. Additionally, variability in cancer type, treatment history, tumor heterogeneity, and the immunosuppressive tumor microenvironment generated due to tumor-intrinsic and tumor-extrinsic factors lead to a failure in response to immune checkpoint inhibitor therapy [4]. The identification of biomarkers including mutational/neoantigen load [9] and the PDL1 expression on tumor and immune cells [10] might predict the responders who would benefit from this therapy, but, in most of the studies, these markers did not show any correlation with the anti-PD1 response [11]. Hence, the concept of combination therapies that can modulate the immunogenicity of tumor cells or can block immunosuppressive TME or target other inhibitory receptors on T-cells comes in place to improve the therapeutic efficiency of checkpoint inhibitors.

The dual checkpoint blockade, using anti-PD1 and anti-CTLA-4 antibodies, was considered a first combinatorial approach in cancer immunotherapy [23, 24]. The outstanding success of the combination of nivolumab (anti-PD1 mAb) and ipilimumab (anti-CTLA-4 mAb) in eliciting an antitumor response in various clinical trials opened the concept of combining immunotherapy with other therapeutic approaches. As a result, various combination immunotherapeutic clinical trials are being conducted nationwide and the outcomes of these studies suggest that these strategies hold the potential to increase the number of patients that might benefit from immunotherapy. Besides CTLA-4 and PD-1, T cells express several inhibitory coreceptors, namely, TIM3, TIGIT, and LAG3 that function as immune checkpoint regulators and can be targeted to activate antitumor immune response. Tim 3 is a negative coinhibitory receptor which negatively regulates T cell responses. Coexpression of TIM3 and PD1 symbols exhausted T cells which leads to loss of function of CD8+ T cells [25, 26] and hence Tim 3 antagonists are suggested as excellent partners for PD1/PDL1 blockade. Another inhibitory receptor expressed on activated CD4 and CD8 T cells is LAG-3 and various studies have suggested that anti-LAG-3 and anti PD-1 treatment cured mice with established colon adenocarcinoma and fibrosarcoma tumors [27]. TIGIT is found on subsets of activated T cells and NK cells are an emerging target in preclinical development. Activation of costimulatory receptors, namely, CD27, 4-1BB, OX40, and GITR, is an alternative approach to activate antitumor immune responses and has recently gained much attention [28]. In addition to inhibitory and costimulatory receptors on T cells, various therapeutic combinations have been emerged which include pairing checkpoint inhibitors with (1) tumor vaccines; (2) IDO inhibitors; (3) oncolytic viruses; (4) inducers of immunogenic cell death; and (5) targeted therapy and various other therapies. Various reviews are available which can provide insight into the combinatorial approaches recently ongoing in clinical trials [29, 30] and will not be discussed here.

## 2. Combinatorial Approaches to Enhance PD1-PDL1 Blockade

**Figure 1:**
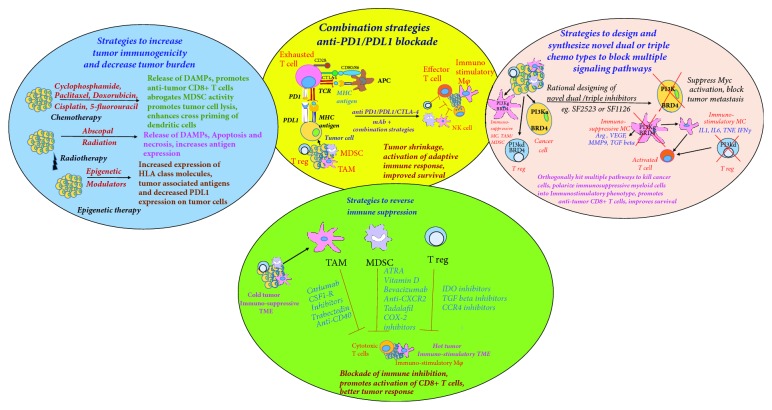
**Combination immunotherapy strategies to reduce tumor burden and to activate durable antitumor immune response**. Left panel shows cartoon of strategies to increase tumor immunogenicity, including certain chemotherapeutic drugs, radiotherapy, and epigenetic modulators which induce immunogenic cell death and release tumor antigens in TME that activate antitumor immune response. Lower panel shows cartoon of agents targeting Tregs, TAMs, and MDSCs, to block immunosuppression, to skew their polarization to proinflammatory state and to promote effector T cell function and to convert cold immunosuppressive TME into hot immune-stimulatory type. Right panel shows novel strategy of rational designing of dual inhibitory chemotypes, e.g., SF2523, dual PI3K/BRD4 inhibitor targeting multiple signaling pathways to kill tumor cells and simultaneously stimulate immune cells to provide durable adaptive immune response. Middle panel shows cartoon of checkpoint inhibitor therapy and pairing of combination therapies with anti-PD1/PDL1 blockade which may significantly improve clinical efficacy of cancer immunotherapy.

In this review, we will focus on strategies that induce tumor immunogenicity and reverse tumor immunosuppression thus increasing antitumor immune responses (Figure 1). We also discuss a novel rational drug design approach to synthesize chemotypes that hit multiple oncogenic pathways in the tumor and reverse myeloid cell-mediated immunosuppression (Figure 1).

### 2.1. Strategies to Increase Tumor Immunogenicity

Chemotherapy and radiotherapy have long been used as conventional methods to reduce tumor burden. It has been recently reported that these therapies activate the immune system by releasing tumor antigens and subverting immunosuppressive factors [31–33]. These facts provide the rationale to explore radiotherapy and chemotherapy with other immunotherapies as these strategic combinatorial approaches could boost the effectiveness of immunotherapy by killing tumor cells and consequently stimulating the immune system and recruit immune cells to the affected area. In this section, we also discuss epigenetic therapy which recently emerged as a new strategy to increase tumor immunogenicity.

#### 2.1.1. Chemotherapy

**Figure 2:**
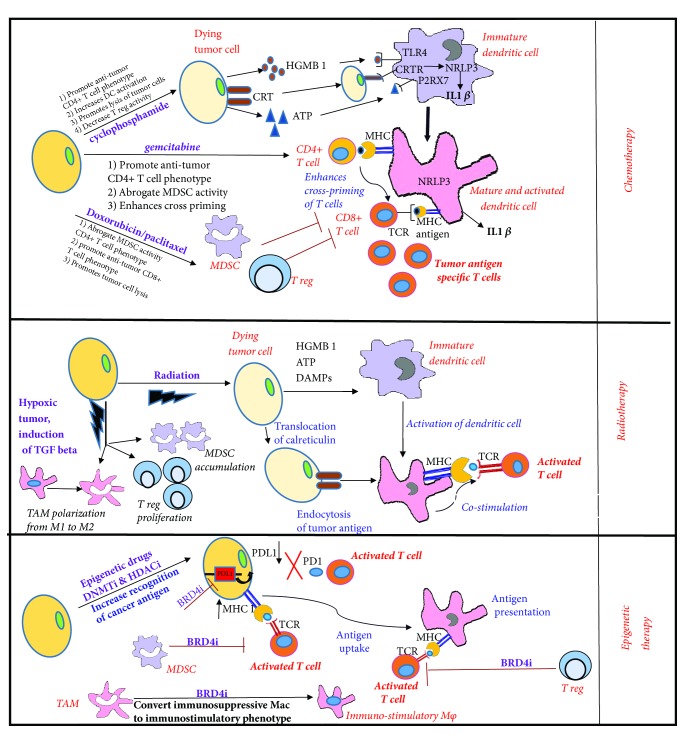
**Mechanisms of inducing immunogenic tumor cell death and decreasing tumor burden by chemotherapy, radiotherapy, and epigenetic therapy.** Upper panel shows how chemotherapeutic drugs induce immunogenic death through release of tumor antigens, secretion of danger associated signals HGMB1 and ATP, and translocation of calreticulin to cell surface. These death-associated molecules bind to TL4R, P2RX7 receptors, and calreticulin receptors which leads to activation of NRLP3 inflammasome and activation of dendritic cells to induce tumor antigen specific T-cell responses. Some chemotherapeutic drugs decrease infiltration and accumulation of Tregs and MDSCs in the TME leading to activation of adaptive immune responses. Middle panel shows the immunogenic cell death induced by radiotherapy. Figure also shows that radiation induces increased transcription of HIF 1*α* and activation of latent TGF beta which leads to increased Treg proliferation and polarization of M1 macrophages to M2 macrophages leading to activation of immunosuppressive TME. Lower panel shows how epigenetic therapy promotes antitumor responses by upregulating tumor-associated antigens and downregulating PDL1 expression. BRD4 inhibitors block accumulation of MDSCs in TME and reduce immunosuppression by promoting the polarization of macrophages into immunostimulatory phenotypes.

Standard chemotherapeutic drugs including paclitaxel, cyclophosphamide, gemcitabine, and cisplatin are reported to modulate tumor immunity at low doses [34]. At their standard dose and schedule, these drugs induce immunogenic cell death (ICD) which involves the release of tumor antigens and the emission of danger signals by dying cancer cells known as DAMPs, which in turn elicit tumor targeting immune responses [34]. These drugs can modulate the immunogenicity of tumor cells through different mechanisms including (1) increasing the expression of tumor antigens and enhancing tumor antigen presentation; (2) upregulating costimulatory molecules (B7-1) and downregulating coinhibitory molecules (B7-H1/PDL1) expressed on the tumor cell surface, which in turn increases effector T-cell function; (3) increasing T-cell mediated lysis of tumor cells through granzyme and perforin-dependent mechanisms; and (4) reducing the infiltration of MDSCs and Tregs in the tumor microenvironment (Figure 2). Various clinical trials of this combination are ongoing to increase the efficacy of immunotherapy and some of which are listed in Table 1.

Preclinical and clinical studies have shown that a low dose of cyclophosphamide or doxorubicin or paclitaxel given one day before vaccination or immunotherapy depleted Tregs, increased IFN gamma secreting CD44+ effector memory T-cells, and enhanced antitumor immune responses in end-stage cancer patients [35, 36]. Likewise, several clinical trials have shown that patients who received a low dose of cyclophosphamide, given 1-3 days before vaccination, showed an increase in effector T-cell function and higher overall survival as compared to those who received the vaccine alone [35, 37]. Based on these reports, a phase 2 clinical study randomized 68 patients with advanced renal cell carcinoma. 33 patients received a low dose of cyclophosphamide 3 days before vaccination with multipeptide vaccine, IMA901 + GM-CSF adjuvant, and 35 patients received vaccination with only IMA901 + GM-CSF adjuvant [38]. In this trial, patients who received 300 mg/m^2^ cyclophosphamide 3 days prior to vaccination with IMA901 + GM-CSF adjuvant had longer overall survival (OS) as compared to the ones who received IMA901 only [38]. Very few reports have examined the efficacy of immune checkpoint blockade with low doses of cyclophosphamide. One study in the cervical cancer model showed that anti-PD1 in combination with a low dose of cyclophosphamide induced infiltration of CD8+ T-cells in the tumor and promoted tumor-free survival [39]. In another study, using the lung adenocarcinoma model, authors showed that the combined use of immunogenic drugs oxaliplatin and cyclophosphamide induced antitumor T-cell immunity and successfully sensitized to checkpoint blockade [40]. These studies led to a clinical trial utilizing cyclophosphamide and pembrolizumab (anti-PD1 mAb) in patients with advanced sarcomas and gastrointestinal stromal tumors [13]. Only 3 out of 50 patients showed clinical benefit in this study and the infiltration of immunosuppressive macrophages and activation of the IDO1 pathway were suggested as reasons for resistance to anti-PD1 therapy in this trial.

Gemcitabine is another example of standard dose chemotherapy which demonstrates potent immunomodulatory effects as it induces apoptosis, enhances cross-priming of CD8+ T-cells, and reduces infiltration of MDSCs in preclinical animal models [41–43]. Gemcitabine given before vaccination or a CD40 agonist augmented the survival of mice in preclinical models [44]. In another study, gemcitabine and cisplatin given after immunotherapy, with an adenoviral vector expressing IFN alpha (Ad.IFN*α*), also demonstrated great antitumor activity compared to chemotherapy or Ad.IFN*α* [45]. These results led to the design of the Telo-Vac study, a Phase 3 trial for patients with advanced or metastatic pancreatic cancer. These studies aimed to study gemcitabine in combination with MHC class 2 telomerase vaccine, GV1001, given with GM-CSF adjuvant [14]. There was a failure in the synergy between immunotherapy and chemotherapy in the Telo-Vac study [14]. In addition to this, other trials have also shown that chemotherapy does not synergize with immunotherapy [46]. A Phase 2 trial integrated pancreas GVAX with standard adjuvant 5-fluorouracil-based chemotherapy in stage 2 and 3 pancreatic cancer patients that led to failure [46]. Contrary to these studies, multiple small trials have shown that concurrent or standard phase chemotherapy may enhance vaccine-induced immunity in various cancers [47]. Recent reports suggest the efficacy of checkpoint inhibitors combined with gemcitabine in the treatment of pancreatic cancer [15]. In 2017, Phase I and II clinical trials were opened to explore gemcitabine with anti-CD40 antibody, APX005M, or with anti-PD-1 mAb nivolumab for pancreatic patients (NCT03214250) [15]. In another clinical trial, gemcitabine was paired with ipilimumab for treating patients with stages 3-4 or recurrent pancreatic cancer that could not be removed by surgery (NCT01473940).

In addition to cyclophosphamide and gemcitabine, other standard chemotherapeutic drugs such as paclitaxel, carboplatin, and dactinomycin have also been combined with immune checkpoint inhibitors for treatment of melanoma, NSCLC, and SCLC [17, 18, 48]. In a recent study, the combination of local chemotherapy (melphalan and dactinomycin) and ipilimumab showed better response rates in patients with advanced melanoma [16]. A Phase 3 trial utilizing the combination of ipilimumab and dacarbazine showed better overall survival response in patients compared to the ones treated with dacarbazine alone [48]. In addition, ipilimumab paired with paclitaxel and carboplatin was evaluated for the treatment of non-small-cell lung cancer (NSCLC) and small-cell lung cancer [17, 18]. For NSCLC, standard progression-free survival (PFS) was observed when patients received phased treatment with chemotherapy followed by ipilimumab. Alternatively, for SCLC, for the same phased treatment, immune-related responses were improved, but no standard PFS was observed. Some of the other clinical trials combining chemotherapy with checkpoint blockade are discussed in Table 1. These trials have shown great promise for treatment of some cancers; however, further investigation of the dosage and schedule of chemotherapeutic drugs is essential for effective translation of these combinatorial approaches in the clinic.

#### 2.1.2. Radiotherapy

Radiotherapy has been known to kill tumors via DNA damage-induced apoptosis or programmed cell death, but recent pieces of evidence suggest that it stimulates tumor antigen release and promotes an immune-mediated antitumor response [49–52]. Contrary to initiating an antitumor immune response, radiation therapy can also promote the generation of immunosuppressive factors that hinder the activation of dendritic cells and impair the function of effector T-cells [53, 54]. Radiotherapy has been reported to promote immunosuppressive tumor microenvironment by (1) increasing transcription of HIF 1 alpha which induces Treg proliferation, MDSC accumulation, and M2 polarization of TAMs and (2) activating latent TGF-beta in the tumor that polarizes TAMs into M2 phenotype and converting CD4+ T-cells into Tregs (Figure 2) [53, 54]. Hence, combining radiotherapy with immune modulators might provide increased benefit to radiation monotherapy.

Radiotherapy is mostly used in combination with surgery or chemotherapy and combination regimens involving radiotherapy and immunotherapies are now emerging treatment regimens [55–58]. These studies suggest that combining radiotherapy with immunotherapy will boost abscopal response rates and will extend the use of radiotherapy in treatment for both local and metastatic disease. The abscopal effect is defined as a phenomenon in the treatment of metastatic cancer whereby localized radiation eradicates distant metastasis and activates systemic antitumor effects. Various preclinical studies have been conducted to explore the role of radiation in combination with immune checkpoint inhibitors [59, 60]. Deng et al. have shown that radiation, in combination with a PD-L1 checkpoint blockade, reduces the infiltration of MDSC in the MC38 colon carcinoma mouse model [59]. In another study, anti-CTLA-4 antibodies combined with radiation showed an abscopal antitumor response in the metastatic 4T1 breast cancer model [60]. In preclinical intracranial glioma models, the combination of radiation and anti-PD1 showed robust systemic immunologic memory in surviving mice and long-term survival in treated mice [61]. Based on the results of these preclinical studies, several clinical trials have been initiated to assess the efficacy of combining radiation therapy with checkpoint inhibitors.

Reports by Postow et al. and Hiniker et al. have shown that melanoma patients treated with the combined regimen of ipilimumab and radiation showed a systemic complete response [62, 63]. In a case report for a patient with NSCLC, concurrent treatment with radiation and ipilimumab led to an abscopal response [64]. In another Phase I/II clinical study, Solvin and his colleagues found that ipilimumab and radiation induced a great response in patients with metastatic, castration-resistant prostate cancer [65]. In a small cohort of patients with skin and kidney cancer, stereotactic body radiotherapy with high dose IL2 showed better tumor response rates over that of IL2 alone [19]. There are several Phase I and 2 clinical trials ongoing in patients with NSCLC for evaluation of radiotherapy with PD-PDL1 immune checkpoint inhibitors, pembrolizumab, and nivolumab [20], outlined in Table 1.

In head and neck cancer, the application of anti-PD1 therapies has shown promising results and, during the last two years, several clinical trials have been initiated to combine immunotherapy with radiation [66], some of which are listed in Table 1. In a randomized Phase 2 clinical trial opened in 2018, pembrolizumab and radiation have been combined for metastatic head and neck squamous cell carcinoma (HNSCC) (NCT03386357). In 2018, another Phase 2 clinical trial was opened to treat locally advanced HNSCC with double checkpoint blockade (durvalumab and tremelimumab) and radiotherapy dependent on intratumoral CD8+ T-cell infiltration (NCT03426657).

#### 2.1.3. Epigenetic Therapy

The epigenetic mechanisms in immune responses have recently gained significant attention because of their potential as therapeutic targets [67–69]. Epigenetic modulators, such as histone deacetylase and DNA methyltransferase (DNMT) inhibitors, increase expression of tumor-associated antigens that lead to improved immunologic recognition of cancer cells and enhanced antitumor response in various tumor models (Figures 1 and 2). HDAC inhibitors, vorinostat and panobinostat, showed robust antitumor activity in colon adenocarcinoma and leukemia model by inducing signs of ICD [70]. HDAC inhibitors are also known to increase expression of HLA class molecules in cancer cells [71] and promote recognition and lysis of cancer cells by activated NK cells [72]. Likewise, DNMT inhibitors are known to promote antitumor responses by upregulating expression of HLA class molecules [73] and tumor-associated antigens [74] in different tumor models. Various studies highlight the combinatorial approach of epigenetic therapy with immune checkpoint blockade in mouse models and human patients as discussed in this review [75]. Epigenetic modulators in combination with checkpoint inhibitors are known (1) to increase T-cell infiltration in TME, (2) to reduce MDSC infiltration in TME, and (3) to enhance surface expression of immune checkpoints [75]. Recently, other epigenetic modulators such as bromodomain inhibitors and histone demethylase inhibitors have also been reported to increase antitumor response. JQ1 is a selective BET/bromodomain inhibitor that is reported to block the interaction between multiple BET proteins (BRD2/3/4) and acetylated histones [76]. Recently JQ1 was reported to suppress PD-L1 expression in ovarian cancer cells, leading to enhancing cytotoxic T-cell responses [77]. Another report by Wang et al. also showed that JQ1 increases the immunogenicity of lymphoma cells by increasing expression of PDL1 [78]. JQ1 has additionally been reported to enhance T-cell persistence and function in various models [79]. JQ1 in combination with anti-PD1 therapy increased antitumor response in a murine model of lung cancer [80]. There are several ongoing clinical trials combining immune checkpoint inhibitors with epigenetic modulators; some of them are shown in Table 1.

### 2.2. Strategies to Reverse Tumor Immunosuppression

The composition of stromal cells in the tumor microenvironment plays an important role in predicting the response of cancer immunotherapy agents. It is now clearly established that tumor-associated macrophages (TAMs), myeloid-derived suppressor cells (MDSCs), and regulatory T-cells (Tregs) are the major mediators of tumor-induced immunosuppression and play a major role in suppressing normal functions of effector T-cells. Hence, they serve as hurdles that limit the therapeutic potential of cancer immunotherapies [81]. These immunosuppressive immune cells limit the activation of CD8+ T-cells through various mechanisms such as (1) blocking T-cell function by the production of anti-inflammatory cytokines, (2) depleting metabolites needed for T-cell proliferation, and (3) engaging with T-cell inhibitory receptors to block cytotoxic T-cell activity. Hence, targeting these immunosuppressive immune cells will increase the efficacy of checkpoint inhibitors.

#### 2.2.1. Macrophage-Targeted Therapy

TAMs are major orchestrators of cancer-related inflammation as they promote tumor growth, angiogenesis, metastasis, tissue remodeling, and immunosuppression. A detailed description of macrophage phenotypes and their function can be found in other reviews [82, 83]. TAM-targeting therapy has recently emerged as a promising novel strategy for the treatment of cancer [84] as these cells possess poor antigen presentation capabilities and suppress the immune response of T-cells by releasing the following factors: arginase, TGF-*β*, and IL10. To overcome the immunosuppressive and protumoral functions of TAMs, current therapies are majorly focused on three aspects: (a) the blockade of macrophage recruitment, (b) the depletion of existing macrophages, and (c) the reprogramming of macrophages into antitumor phenotypes. In this section, we also discuss toll-like receptor agonists in clinical trials and their potential pairing with checkpoint inhibitors.

*(a) Blockade of Recruitment of Macrophages in the Tumor Microenvironment. *Chemokines have long been known to promote the infiltration of myeloid cells in the tumor microenvironment [85]. Chemokine-cytokine mediated infiltration of macrophages can be blocked by targeting CCR2 receptors that bind to ligands, CCL2, CCL8, and CCL16. Inhibition of CCL2 with specific antibodies reduced tumor growth in different experimental models such as prostrate, melanoma, breast, liver, and lung cancer [86–88]. In the Phase I clinical trial, administration of anti-CCL2 IgG1*κ* mAb, carlumab (CNTO 888), was well tolerated and showed greater efficacy in patients with solid tumors, while there was no response of this antibody in the Phase II clinical trial that involved patients with castration-resistant prostate cancer [21, 22]. Few preclinical studies demonstrate enhanced antitumor responses when carlumab was administered in combination with chemotherapeutic drugs [86]; however, combining carlumab with a chemotherapy regimen did not show the significant antitumor immune response in a clinical trial conducted on patients with solid tumors [89]. By contrast, the combination of novel CCR2 antagonist, PF-04136309, with cytotoxic cocktail, FOLFIRINOX, showed an improved antitumor response in a clinical trial conducted on pancreatic adenocarcinoma patients (NCT01413022) [90]. There is only one report illustrating that the blockade of CCL2 enhanced the immunotherapeutic effect of anti-PD1 in lung cancer [91]. However, therapeutic efficacy of this combination has yet to be tested in clinical trials.

*(b) Reduction of TAMs by Depleting TAMs or Their Precursors. *CSF-1 receptor is expressed by most of the cells of the monocytic lineage and is a direct target to block monocytic precursors directly and indirectly. CSF-1, commonly known as MCSF, is used as a differentiation factor for cells of monocyte or macrophage lineages. Antagonists or antibodies to CSF1R have been developed and tested in various preclinical models (e.g., cervical cancer, pancreatic cancer, and glioblastoma) in combination with chemotherapy, radiation therapy, and checkpoint inhibitors whereby they depleted immunosuppressive macrophages and increased the CD8/CD4 ratio in the tumors [92]. Clinical trials combining the use of CSF1-R inhibitors and chemotherapy have been initiated for the treatment of various cancers based on initial preclinical data in mouse breast cancer models where a CSF1R blockade increased the efficiency of paclitaxel [93]. RG7155 (Emactuzumab) is a humanized monoclonal antibody that blocks CSF1R activation. A Phase I clinical trial of RG7155 in patients with diffuse-type giant cell tumors (Dt-GCT) showed measurable clinical responses, with the disintegration of tumor mass in one patient. This data is also correlated with the depletion of TAMs from tumor biopsies and a substantial increase in CD8+ T-cell infiltration [94]. Pexidartinib, an inhibitor of CSF-1R, augmented antitumor immune responses when combined with radiotherapy in glioblastoma models [95]. BLZ 945, another potent and selective inhibitor of CSF-1R, blocked recruitment of bone marrow-derived macrophages and tumor progression in glioma models [96]. Currently, there are several ongoing clinical trials to combine these CSF1-R inhibitors with PD-1/PD-L1 inhibitors as listed in Table 1.

Trabectedin, which was originally recognized for its ability to induce cell cycle arrest and death, was found to cause a partial depletion of circulating monocytes and TAMs in cancer patients [97]. Trabectedin-induced TAM reduction was associated with decreased angiogenesis in murine tumors and human sarcomas [98]. Based on the efficiency of trabectedin in the selective killing of mononuclear phagocytes from tumors in preclinical mouse models, it has been translated to numerous clinical trials either as a single agent or in combination with other drugs to study safety and efficacy in human tumors. To date, two Phase III clinical trials, mentioned in Table 1, have confirmed the therapeutic advantage of trabectedin. These observations open up the option to combine trabectedin with checkpoint blockade inhibitors.

*(c) Reprogramming of Macrophages into Antitumor Phenotype. *Macrophages are highly plastic cells and can change their phenotype in accordance with environmental cues. In response to various signals produced by tumor and stromal cells, proinflammatory macrophages shift to an immunosuppressive phenotype that blocks antitumor immunity [99]. Hence, targeting the molecular pathways/signaling nodes in the macrophages that regulate the transition of protumorigenic MΘs into antitumorigenic MΘs will activate the immune response in cancer [100]. Various preclinical studies identified signaling pathways or key genes such as the jumonji domain, containing proteins (JMJD3), STAT3, STAT6, Myc, Rac2, PI3K*γ*, Btk, etc. which play a crucial role in stimulating alternative activation of macrophages and promoting tumor growth [101–105]. Recent studies have provided evidence that targeting PI3K*γ* in macrophages can polarize macrophages into immunostimulatory phenotypes, promote CD8+ T-cell cytotoxicity, and augment the tumor suppressive effects of anti-PD1 antibody in mouse models [101, 103, 106]. These studies have led to the clinical trial of PI3K*γ* inhibitor IPI549 in combination with nivolumab for advanced solid tumors [106, 107]. In another preclinical study in pancreatic cancer model, Bruton's tyrosine kinase (BTK) inhibitor ibrutinib repolarized macrophages into the M1 phenotype and promoted cytotoxic CD8+ T-cell activity [108]. This strategy is under consideration for the opening of a clinical trial to study ibrutinib in combination with checkpoint inhibitors for pancreatic cancers [108].

Another example of macrophage targeting comes from the administration of the anti-CD40 antibody in a preclinical model of pancreatic cancer. This study showed that use of the anti-CD40 antibody repolarized M2-like macrophages into an M1-like phenotype leading to a reduction in tumor growth [109]. This preclinical data lead to the clinical trial of human CD40 agonist with gemcitabine in advanced pancreatic cancer patients which showed partial responses [109]. RO7009789 is another CD40 agonist which has been tested in combination with chemotherapy and immunotherapy in solid tumors and are listed in Table 1.

*(d) Toll-Like Receptor Agonists. *Toll-like receptors (TLRs), as the most important pattern-recognition receptors in innate immunity, play a critical role in the defense against infection and disease including cancer. TLRs are expressed in a wide range of immune cells including monocytes, macrophages, and dendritic cells. Certain TLRs have been shown to enhance DC maturation and antigen presentation leading to effective antitumor effects. Thus, the agonists of TLR signaling are explored as anticancer agents or vaccines to induce effective immune reactions against tumor antigens. A study by Bald et al. has shown that TLR-3 activation induces type I interferon and synergizes with anti-PD1 therapy in the melanoma model [110]. Poly-IC-LC, a TLR 3 ligand, has been used in combination with pembrolizumab for the treatment of colon cancer (NCT02834052). Furthermore, TLR9 agonists are reported to increase T-cell infiltration and to induce CD4 and CD8 T-cell antitumor immunity in various mouse models [111]. In a lymphoma mouse model, a TLR9 agonist (intratumoral CpG) in combination with anti-OX40 and anti-CTLA-4 cured large and systemic lymphoma tumors without the need for chemotherapy [112]. A recent report by Sato-Kaneko et al. suggests that TLR7 and TLR9 agonists in combination with anti-PD1 mAb increased M1 to M2 macrophage ratio and increased infiltration of IFN gamma secreting CD8+ T-cells in head and neck cancer models [113]. There are several ongoing clinical trials to test the combination of TLR9 agonist with anti-PD1 therapy in various cancers. DV281, a TLR9 agonist, has been used in combination with anti-PD1 antibody in Phase I clinical trial including NSCLC patients (NCT03326752).

#### 2.2.2. MDSCs Targeted Therapy

Myeloid-derived suppressor cells are immature myeloid cells that expand in pathophysiological conditions like inflammation and cancer and suppress T-cell activity [114, 115]. They play an important role in mediating immunosuppression and are considered a promising target to be paired with check point blockade. Similar to macrophage targeted therapy, MDSC targeted therapy can also be divided into three sections based on the strategies to block its accumulation, recruitment, and reversal of MDSC-mediated immunosuppression.

*(a) Blocking Accumulation of MDSCs and Their Differentiation into Macrophages or Dendritic Cells. *In order to reduce the accumulation of MDSCs, the process of myelopoiesis has to be normalized and the strategies to differentiate them into normal macrophages or dendritic cells has to be stimulated. Various studies have suggested that a blockade of retinoic acid signal transduction by all-trans-retinoic acid (ATRA) promotes the differentiation of MDSCs into macrophages and dendritic cells in mouse and human samples [116]. ATRA is a vitamin A derivative and is an FDA approved drug for the treatment of acute promyelocytic leukemia (APL) [117]. ATRA has been applied to two clinical trials, in combination with immunotherapy, to target MDSCs in cancer patients. The first clinical trial was in patients with metastatic renal cancer [118] and the second trial was in patients with small lung cancer [119]. Both trials resulted in reductions of MDSC accumulation and improved patient survival. In another Phase 2 clinical trial, ATRA was combined with ipilimumab to treat stage IV melanoma patients (NCT02403778). Similar to ATRA, vitamin D3 differentiates immature MDSCs into macrophages and dendritic cells. A small clinical study involving 17 HNSCC patients showed that treatment with vitamin D3 reduced the number of infiltrating MDSCs, increased the number of mature tumor-infiltrating dendritic cells, and an improved antitumor immune response [120].

Recent studies have shown that anti-VEGF antibody, bevacizumab—which is used to treat VEGF-mediated angiogenesis, can also be used to decrease circulating immature myeloid cells in cancer patients. VEGF is a key mediator of tumor-induced angiogenesis [121] and plays an important role in inhibiting the maturation of myeloid cells [122]. A small clinical trial involving 19 metastatic, colorectal patients showed that bevacizumab decreased immature MDSCs and improved the stimulatory capacity of DCs isolated from treated patients [123]. Based on this clinical trial, Phase I and 2 clinical trials enrolled 62 patients with advanced renal cell carcinoma for treatment with bevacizumab, entinostat, and atezolizumab.

*(b) Blockade of Recruitment of MDSCs. *MDSCs are recruited in the tumor site to promote immunosuppression and this process is mediated by chemokines that have been secreted by chemokine receptors found on MDSCs in the tumor microenvironment. The roles of CCL2, CCL5, CCL7, and CXCL8 and their receptors, CCR2 and CCR4, in the recruitment of MDSCs are well described [124]. A study by Highfill et al. has shown that anti-CXCR2 plus anti-PD-1 mAb blocked MDSC recruitment and enhanced anti-PD1 efficacy [124]. In another study, the combination of a CCR1 inhibitor (CCX9588) and PD-L1 inhibitor increased the antitumor effects in a murine model of breast cancer [125]. Yet another clinical trial in melanoma patients is utilizing the combination of pembrolizumab with MDSC targeting by SX-682, a small molecule dual inhibitor of C-X-C-motif chemokine ligands 1 and 2 (NCT03161431). RTA-408, an anti-inflammatory drug, is also explored within melanoma patients.

*(c) Reversal of MDSC Mediated Immunosuppression. *Phosphodiesterase-5 inhibitors are known to inhibit the immunosuppressive function of MDSCs by decreasing IL-4*α* expression. There are three PDE-5 inhibitors in clinical trials: sildenafil, tadalafil, and vardenafil. In preclinical mouse models, sildenafil has reduced tumor growth by blocking the immunosuppressive function of MDSCs and enhanced the activation and infiltration of T-cells [126, 127]. Tadalafil has been applied to patients with head and neck squamous carcinoma and melanoma where it showed improved clinical outcome due to a reduction in tumor-infiltrating MDSCs [128, 129].

Recent studies have shown that PI3K*γ* plays an important role in the trafficking of MDSCs and their polarization into an immunosuppressive phenotype [101, 130]. PI3K*δ*/*γ* inhibitor, IPI-145, in combination with anti-PDL1 antibodies resulted in the inhibition of MDSC activity, increased CD8+ T-cell infiltration, and improved survival in head and neck tumor models [131].

#### 2.2.3. Treg Targeted Therapy

Tregs are highly immunosuppressive fractions of CD4+ T-cells and are known to play a major role in maintaining self-tolerance. Treg cells exhibit their suppressive activity via several mechanisms including inhibition of APC maturation through the CTLA-4 pathway; secretion of inhibitory cytokines such as IL10, TGF beta, and IL35; and expression of granzyme and perforin which kills effector T-cells. There are several potential therapies that target Treg cell suppression either directly or indirectly including candidates targeting CD25, CTLA-4, OX-40, GITR, and CCR4. In this section, we will discuss IDO inhibitors and TGF beta inhibitors.

*(a) CCR4 Inhibitors. *It has been previously reported that tumor cells and macrophages produce CCL22 which chemotracts Treg cells expressing the CCR4 receptor. This antibody is reported to deplete CD25+ Tregs and promote antitumor immune responses in human patients [132]. Hence, anti-CCR4 is an attractive target to combine with checkpoint inhibitors. Currently, the anti-CCR4 antibody is being tested in combination with nivolumab (NCT02705105) or durvalumab (NCT02301130) in patients with advanced solid tumors.

*(b) IDO Inhibitors. *IDO is a metabolic enzyme which catalyzes the cleavage of L-tryptophan to its metabolites, resulting in the generation of Kynurenine [133]. IDO is expressed by tumor cells and MDSCs in response to interferon gamma [134]. Recent studies have provided evidence that IDO activity is critical to support the activity of FoxP3 Tregs [135] and MDSCs [136], leading to suppression of the activity of T-cells and NK cells [137]. IDO has been reported to promote T-cell resistance to anti-CTLA 4 therapy in murine melanoma models [138]. Recent reports have shown that combined inhibition of IDO and immune checkpoint inhibitors (CTLA-4, PD1, and PD-L1) synergize in melanoma mouse models. Based on the impressive preclinical data in melanoma model, several IDO inhibitors are explored in combination with CTLA-4 and PD-1 inhibition [139]. There are several IDO inhibitors in a clinical trial with epacadostat (INCB024360), which has been explored in clinical trials mostly in combination with immune checkpoint inhibitors. A Phase I trial combining epacadostat and ipilimumab (anti-CTLA-4) was well tolerated in patients of metastatic melanoma [140]. Pembrolizumab and the epacadostat were recently reported to show promising response rates in NSCLC and melanoma, which lead to a Phase III clinical trial of this combination in melanoma patients [141]. Very recently, this Phase III trial has been expanded to NSCLC; head and neck cancer; and renal and bladder cancers [142]. Indoximod is another IDO inhibitor in clinical trials [143]. A Phase 2 study combining indoximod with ipilimumab or anti-PD1 (pembrolizumab or nivolumab) is currently ongoing (NCT02073123).

*(c) TGFβ Inhibitors. *Transforming growth factor-*β* (TGF*β*) is a cytokine that plays a crucial role in mediating immunosuppression in the tumor microenvironment by stimulating Tregs [144]. A study in preclinical melanoma models (BRAFV600EPTEN−/−) has shown synergy combining TGF-*β* receptor kinase inhibitor I with anti-CTLA-4 antibodies [145]. Recently, clinical trials have been ongoing to test the combination of TGF-*β* inhibitor (galunisertib) [146] and PD-1/L1 checkpoint blockade (durvalumab or nivolumab) in patients with pancreatic cancer (NCT02734160) and hepatocellular carcinoma; NSCLC; and glioblastoma (NCT02423343).

### 2.3. Strategies to Design and Synthesize Novel Dual or Triple Inhibitory Chemotypes That Can Block Multiple Pathways

Concomitant inhibition of multiple pathways required for tumor progression has recently been established as an innovative strategy to improve targeted therapies in cancer [147–151]. The “dual- or triple-targeted single agent” strategy could provide similar benefits as multiple combination therapies do, with minimal convolutions observed in combination approach including drug toxicities, lengthy clinical investigations, and high treatment costs. SF1126 is the first PI3K/BRD4 inhibitor reported by SignalRx Pharmaceuticals, Inc., blocking tumor growth in various preclinical mouse models [152–155]. SF1126 blocked tumor angiogenesis and metastasis, as well as increasing the M1 to M2 ratio in various preclinical mouse models [101]. Based on the promising preclinical results, various clinical trials of SF1126 were opened. A Phase I clinical trial of SF1126 enrolled 44 patients with advanced solid tumors and B cell malignancies at 9 dose levels (90-1110 mg/m^2^/day) (155). SF1126 was well tolerated in patients with stable disease (SD) as the best response in patients with advanced malignancies. Based on this convincing data, Phase 1 clinical trials of SF1126 were opened for patients with relapsed or refractory neuroblastoma (NCT02337309) or those with advanced hepatocellular carcinoma (NCT03059147). The combination of SF1126 with anti-PD1/PDL1 inhibitors is in the planning stages for treating advanced solid tumors. In addition to SF1126, various other multikinase inhibitors such as BI-2536 (clinical PLK1) and TG-101348 (JAK2/FLT3 kinase inhibitor) are reported to have BRD4 activity and are suggested as dual kinase/bromodomain inhibitors [156]. Another report by Weinstock et al. has demonstrated the selective dual inhibition of cancer-related deubiquitinating proteases, USP7 and USP47, and has shown* in vivo* activity against multiple myeloma and B cell leukemia [149].

Morales et al. have recently reported the synthesis of a 5-morpholino-7H-thieno [3,2-b] pyran-7-one (TP scaffold) system to design novel inhibitors having improved potency towards PI-3K [157]. SF2523 is a dual PI3K/BRD4 inhibitor generated from the TP series, which orthogonally hit PI3K and BRD4 to block expression, activation, and stabilization of Myc, leading to reduced tumor growth and metastasis in various preclinical models [158–160]. Unpublished results by Joshi et al. suggest that SF2523 is an immunooncological inhibitor that hits tumor cells, increases the ratio of M1 to M2, and increases the infiltration of cytotoxic CD8+ T-cells in various tumor models. This molecule has PI3K*α* and BRD4 activity which blocks tumor growth by inducing apoptosis and cell cycle arrest, suppressing PDL1 expression on tumor cells; and simultaneously this molecule blocks myeloid cell-derived immunosuppression and provides a durable antitumor immune response in various tumor models due to its potent PI3K*γ* and PI3K*δ* activity. Using this TP scaffold and computational methods, novel dual or triple PI3K/BRD4/CDK4/6, PI3K/PARP, PI3K/BRD4/HDAC, PI3K/MEK, and PI3K/IDO inhibitory chemotypes are engineered by SignalRx Pharmaceuticals [161]. These rationally designed chemotypes are highly selective and potent against their targets. SRX3177 is a “first in class” triple PI3K-BRD4-CDK4/6 inhibitor, for combinatorial inhibition of three oncogenes promoting cancer cell growth: phosphatidylinositol-3 kinase (PI3K), cyclin-dependent kinases 4 and 6 (CDK4/6), and the epigenetic regulator BRD4 [162] in a single molecule. This chemotype showed potent* in vitro* and* in vivo* activity in mantle cell lymphoma, neuroblastoma, and hepatocellular carcinoma tumor models [162]. Another report suggests the development of rationally designed, pazopanib based, HDAC and VEGFR, dual inhibitors that target cancer epigenetics and angiogenesis simultaneously, and this chemotype has shown antitumor efficacy in human colorectal carcinoma models [150]. This rational drug design strategy has recently gained significant attention resulting in a number of dual or triple inhibitory chemotypes for the treatment of cancer. This strategy of rationally designing novel dual or triple chemotypes which can effectively kill tumor cells and, at the same time, activate immune cells will be an effective combination regimen to explore with immunotherapy. Although most of these chemotypes are not yet explored in clinical trials, there is strong hope that these rationally designed chemotypes will show a durable antitumor immune response when combined with immunotherapy.

## 3. Conclusions

In summary, the durable antitumor response generated by the application of checkpoint inhibitors has revolutionized the field of cancer immunotherapy. Very soon it will be realized that the clinical benefit obtained from the immune checkpoint blockade is limited to only a small subset of patients and that most patients do not respond to this therapy. The combination of immune checkpoint blockade with several other anticancer treatments has shown remarkable success in various cancers and provided hope for the patients who do not respond to checkpoint inhibitor monotherapy. We have summarized the therapeutic potential of checkpoint inhibitors with drugs that increase tumor immunogenicity, reduce tumor burden, and reverse tumor-mediated immunosuppression, leading to an effective and durable antitumor immune response. In the end, we provided a novel approach to rationally design dual or triple inhibitory chemotypes that can concomitantly hit several tumor-promoting pathways and increase the immune effector response by blocking myeloid cell-mediated tumor immunosuppression. This novel approach is early in its development stage, but the promising antitumor results generated so far by use of dual or triple inhibitor chemotypes in different cancer models provide a rationale to continue exploring these agents with immune checkpoint inhibitors.

## Figures and Tables

**Table 1:** Clinical studies of combination strategies to improve immunotherapy.

**Combinatorial approach**		**Combination Therapy & Drug Used**	**Cancer Vaccine or Immunotherapy** **Drug Used**	**Tumor Type & Reference**
***Strategies to increase tumor immunogenicity***	***Chemotherapy***	Cyclophosphamide	Five peptide cancer vaccine	Phase I clinical trial in advanced solid tumors (NCT00676949) [12]
			Pembrolizumab	Advanced sarcomas and gastrointestinal tumors (NCT02406781) [13]
		Gemcitabine	GV1001 vaccine given with GMCSF adjuvant	In Phase 3 Telo-Vac clinical study in pancreatic cancer [14].
			Anti-CD40 antibody APX005M or with nivolumab	Phase I and II clinical trial for pancreatic patients (NCT03214250) [15]
			Ipilimumab	Phase I clinical trial for Pancreatic Cancer (NCT01473940)
		Melphalan and Dactinomycin	Ipilimumab	Phase 2 trial in advanced melanoma (NCT01323517)
		Dacarbazine	Ipilimumab	Phase 3 clinical trial in advanced melanoma (NCT00324155) [16]
		Paclitaxel and Carboplatin	Ipilimumab	Phase 2 clinical trial for non–small cell lung cancer (NSCLC) (NCT00527735) and small-cell lung cancer (NCT 00527735) [17, 18]
		Paclitaxel, Carboplatin and Premetrexed	Pembrolizumab	Phase 2 clinical trial for lung cancer (NCT02039674)
		Paclitaxel, Carboplatin, gemcitabine	Nivolumab	Phase I clinical trial for Pancreatic, breast, NSCLC (NCT02309177)
	***Radiotherapy***	Stereotactic body radiation	Ipilimumab	Melanoma (NCT01970527), prostrate (NCT00323882) and lung cancer (NCT02239900).
			High dose IL-2	Kidney and skin cancer (NCT01896271) [19]
			Pembrolizumab and nivolumab	Phase I and II clinical trials of radiation with in NSCLC (NCT3148327) [20]
			Nivolumab	Glioblastoma (NCT02617589), triple negative breast cancer (NCT02499367)
			Pembrolizumab	metastatic Head and neck squamous carcinoma (NCT03386357)
			Durvalumab and tremelimumab	Locally advanced Head and neck squamous carcinoma NCT03426657
	***Epigenetic Therapy***	SGI-110 (DNMT inhibitor)	Ipilimumab	Melanoma (NCT02608437)
		Vorinostat (HDAC inhibitor)	Pembrolizumab	Renal cell carcinoma (NCT02619253)
		Entinostat (HDAC inhibitor)	Pembrolizumab	Melanoma and NSCLC (NCT02437136)
		Azacitidine (DNMT inhibitor) + entinostat	Nivolumab	NSCLC (NCT01928576)

**** ***Strategies to reverse tumor immunosuppression***	***Macrophage Targeted Therapy*** *Blocking recruitment of macrophages*	Carlumab (human anti-CCL2 IgG1*κ* mAb (CNTO 888)		Prostrate cancer [21] and advanced cancer patients [22]
		PF-04136309 (CCL2-CCR2 axis inhibitor)		PF-04136309 plus FOLFIRINOX show better treatment response in patients with locally advance PDAC (NCT01413022)
	*Reduction of TAMs by depleting TAMs or its precursor*	RG7155 (anti-CSF1R neutralizing antibodies)		RG7155 demonstrated therapeutic efficacy in patients with diffuse-type giant cell tumor
				Plexiform Neurofibromas, Neurofibromatosis (NCT02390752)
		PLX3397 (pexidartinib) (CSF-1R inhibitor)	Urvalumab	Advanced Pancreatic and colorectal cancer (NCT02777710)
			Pembrolizumab	Melanoma and solid tumors (NCT02452424)
		BLZ945 (CSFIR inhibitor)	PDR001 (anti-PD1)	Phase I/II clinical trial in advanced solid tumors (NCT02829723)
		Trabectedin		Phase III study showed trabectedin plus PLD improves overall survival than PLD alone in ovarian cancer Phase III trial in locally advanced or metastatic liposarcoma (NCT01692678)
	*Reprogramming of macrophages into anti-tumor phenotype*	IPI-549	Pembrolizumab	Solid tumors (NCT02637531)
		RO7009789 (Agonist CD40 Ab)	Nab-paclitaxel and gemcitabine	Pancreatic adenocarcinoma (NCT02588443)
			Atezolizumab	Metastatic solid tumors (NCT02304393)
	*TLR agonists*	*DV821 (TLR9 agonist)*	Anti-PD1 ab	Phase I NSCLC NCT03326752
		Poly-IC-LC (TLR3)	Pembrolizumab	Colon cancer (NCT02834052)
	***MDSC Targeted therapy*** *Reduction in accumulation of MDSCs*	ATRA	Ipilimumab	Stage IV melanoma (NCT02403778)
		Bevacizumab, entinostat	Atezolizumab	Advanced renal cell carcinoma (NCT03024437)
	*Blockade or recruitment of MDSCs*	SX-682	Pembrolizumab	Melanoma (NCT03161431)
		RTA-408	Ipilimumab Nivolumab	Melanoma (NCT02259231)
	***Treg targeted therapy***	CCR4 inhibitor	Pembrolizumab	Phase 1 and 2 for solid tumors (NCT02178722)
		Epacadostat (IDO inhibitor)	Durvalumab	Advanced solid tumors (NCT02318277)
			Atezolizumab	NSCLC (NCT02298153)
		galunisertib (TGF beta inhibitor)	Durvalumab or nivolumab	Pancreatic cancer (NCT02423343).

***Strategies to design and synthesize novel dual or triple inhibitory chemotypes that can block multiple pathways***		SF1126		Solid tumors (NCT00907205)
				Recurrent or progressive SCCHN tumors (NCT02644122)
				Neuroblastoma (NCT02337309)
			Nivolumab	Hepatocellular carcinoma (NCT03059147)

## References

[1] Hodi F. S., O'Day S. J., McDermott D. F. (2010). Improved survival with ipilimumab in patients with metastatic melanoma. *The New England Journal of Medicine*.

[2] Topalian S. L., Hodi F. S., Brahmer J. R. (2012). Safety, activity, and immune correlates of anti-PD-1 antibody in cancer. *The New England Journal of Medicine*.

[3] Brahmer J. R., Tykodi S. S., Chow L. Q. M. (2012). Safety and activity of anti-PD-L1 antibody in patients with advanced cancer. *The New England Journal of Medicine*.

[4] Pitt J. M., Vétizou M., Daillère R. (2016). Resistance Mechanisms to Immune-Checkpoint Blockade in Cancer: Tumor-Intrinsic and -Extrinsic Factors. *Immunity*.

[5] O'Donnell J. S., Long G. V., Scolyer R. A., Teng M. W., Smyth M. J. (2017). Resistance to PD1/PDL1 checkpoint inhibition. *Cancer Treatment Reviews*.

[6] Jenkins R. W., Barbie D. A., Flaherty K. T. (2018). Mechanisms of resistance to immune checkpoint inhibitors. *British Journal of Cancer*.

[7] Sharma P., Hu-Lieskovan S., Wargo J. A., Ribas A. (2017). Primary, Adaptive, and Acquired Resistance to Cancer Immunotherapy. *Cell*.

[8] Marincola F. M., Jaffee E. M., Hicklin D. J., Ferrone S. (2000). Escape of human solid tumors from T-cell recognition: molecular mechanisms and functional significance. *Advances in Immunology*.

[9] Rizvi N. A., Hellmann M. D., Snyder A. (2015). Mutational landscape determines sensitivity to PD-1 blockade in non-small cell lung cancer. *Science*.

[10] Garon E. B., Rizvi N. A., Hui R. (2015). Pembrolizumab for the treatment of non-small-cell lung cancer. *The New England Journal of Medicine*.

[11] Carbognin L., Pilotto S., Milella M. (2015). Differential activity of nivolumab, pembrolizumab and MPDL3280A according to the tumor expression of programmed death-ligand-1 (PD-L1): sensitivity analysis of trials in melanoma, lung and genitourinary cancers. *PLoS ONE*.

[12] Murahashi M., Hijikata Y., Yamada K. (2016). Phase I clinical trial of a five-peptide cancer vaccine combined with cyclophosphamide in advanced solid tumors. *Clinical Immunology*.

[13] Toulmonde M., Penel N., Adam J. (2017). Combination of pembrolizumab and metronomic cyclophosphamide in patients with advanced sarcomas and GIST: A French Sarcoma Group phase II trial.. *Journal of Clinical Oncology*.

[14] Middleton G., Silcocks P., Cox T. (2014). Gemcitabine and capecitabine with or without telomerase peptide vaccine GV1001 in patients with locally advanced or metastatic pancreatic cancer (TeloVac): an open-label, randomised, phase 3 trial. *The Lancet Oncology*.

[15] Immunotherapy PIfC Evaluating the Safety and Efficacy of a CD40 Antibody, Anti-PD-1 Checkpoint Inhibitor and Chemotherapy in Combination, Pancreatic Cancer Clinical Trial Combining Immunotherapy and Chemotherapy, 2017. https://www.parkerici.org/research_project/cd40-pancreatic-cancer-clinical-trial-immunotherapy-and-chemotherapy/.

[16] Ariyan C. E., Brady M. S., Siegelbaum R. H. (2018). Robust antitumor responses result from local chemotherapy and CTLA-4 Blockade. *Cancer Immunology Research*.

[17] Lynch T. J., Bondarenko I., Luft A. (2012). Ipilimumab in combination with paclitaxel and carboplatin as first-line treatment in stage IIIB/IV non-small-cell lung cancer: results from a randomized, double-blind, multicenter phase II study. *Journal of Clinical Oncology*.

[18] Reck M., Bondarenko I., Luft A. (2013). Ipilimumab in combination with paclitaxel and carboplatin as first-line therapy in extensivedisease-small-cell lungcancer: Results from a randomized, double-blind, multicenter phase 2 trial. *Annals of Oncology*.

[19] Seung S. K., Curti B. D., Crittenden M. (2012). Phase 1 study of stereotactic body radiotherapy and interleukin-2: Tumor and immunological responses. *Science Translational Medicine*.

[20] Hanna G. G., Illidge T. (2016). Radiotherapy and Immunotherapy Combinations in Non-small Cell Lung Cancer: A Promising Future?. *Clinical Oncology*.

[21] Pienta K. J., Machiels J.-P., Schrijvers D. (2013). Phase 2 study of carlumab (CNTO 888), a human monoclonal antibody against CC-chemokine ligand 2 (CCL2), in metastatic castration-resistant prostate cancer. *Investigational New Drugs*.

[22] Sandhu S. K., Papadopoulos K., Fong P. C. (2013). A first-in-human, first-in-class, phase i study of carlumab (CNTO 888), a human monoclonal antibody against CC-chemokine ligand 2 in patients with solid tumors. *Cancer Chemotherapy and Pharmacology*.

[23] Wolchok J. D., Kluger H., Callahan M. K. (2013). Nivolumab plus Ipilimumab in advanced melanoma. *The New England Journal of Medicine*.

[24] Curran M. A., Montalvo W., Yagita H., Allison J. P. (2010). PD-1 and CTLA-4 combination blockade expands infiltrating T cells and reduces regulatory T and myeloid cells within B16 melanoma tumors. *Proceedings of the National Acadamy of Sciences of the United States of America*.

[25] Zhou Q., Munger M. E., Veenstra R. G. (2011). Coexpression of Tim-3 and PD-1 identifies a CD8^+^ T-cell exhaustion phenotype in mice with disseminated acute myelogenous leukemia. *Blood*.

[26] Fourcade J., Sun Z., Benallaoua M. (2010). Upregulation of Tim-3 and PD-1 expression is associated with tumor antigen-specific CD8^+^ T cell dysfunction in melanoma patients. *The Journal of Experimental Medicine*.

[27] Woo S.-R., Turnis M. E., Goldberg M. V. (2012). Immune inhibitory molecules LAG-3 and PD-1 synergistically regulate T-cell function to promote tumoral immune escape. *Cancer Research*.

[28] Chen L., Flies D. B. (2013). Molecular mechanisms of T cell co-stimulation and co-inhibition. *Nature Reviews Immunology*.

[29] Vilgelm A. E., Johnson D. B., Richmond A. (2016). Combinatorial approach to cancer immunotherapy: Strength in numbers. *Journal of Leukocyte Biology*.

[30] Ott P. A., Hodi F. S., Kaufman H. L., Wigginton J. M., Wolchok J. D. (2016). Combination immunotherapy: A road map. *Journal for ImmunoTherapy of Cancer*.

[31] Bracci L., Schiavoni G., Sistigu A., Belardelli F. (2014). Immune-based mechanisms of cytotoxic chemotherapy: implications for the design of novel and rationale-based combined treatments against cancer. *Cell Death & Differentiation*.

[32] Zitvogel L., Kepp O., Kroemer G. (2011). Immune parameters affecting the efficacy of chemotherapeutic regimens. *Nature Reviews Clinical Oncology*.

[33] Takeshima T., Chamoto K., Wakita D. (2010). Local radiation therapy inhibits tumor growth through the generation of tumor-specific CTL: Its potentiation by combination with TH1 cell therapy. *Cancer Research*.

[34] Emens L. A., Middleton G. (2015). The interplay of immunotherapy and chemotherapy: harnessing potential synergies. *Cancer Immunology Research*.

[35] Ghiringhelli F., Menard C., Puig P. E. (2007). Metronomic cyclophosphamide regimen selectively depletes CD4^+^CD25^+^ regulatory T cells and restores T and NK effector functions in end stage cancer patients. *Cancer Immunology, Immunotherapy*.

[36] Machiels J.-P. H., Todd Reilly R. T., Emens L. A. (2001). Cyclophosphamide, doxorubicin, and paclitaxel enhance the antitumor immune response of granulocyte/macrophage-colony stimulating factor-secreting whole-cell vaccines in HER-2/neu tolerized mice. *Cancer Research*.

[37] Emens L. A., Jaffee E. M. (2003). Enhancement of an Allogeneic GM-CSF-Secreting Breast Cancer Vaccine by Immunomodulatory Doses of Cyclophosphamide and Doxorubicin. *Defense Technical Information Center*.

[38] Walter S., Weinschenk T., Stenzl A., Zdrojowy R., Pluzanska A., Szczylik C. (Aug 2012). Multipeptide immune response to cancer vaccine IMA901 after single-dose cyclophosphamide associates with longer patient survival. *Nature Medicine*.

[39] Mkrtichyan M., Najjar Y. G., Raulfs E. C. (2011). Anti-PD-1 synergizes with cyclophosphamide to induce potent anti-tumor vaccine effects through novel mechanisms. *European Journal of Immunology*.

[40] Pfirschke C., Engblom C., Rickelt S. (2016). Immunogenic Chemotherapy Sensitizes Tumors to Checkpoint Blockade Therapy. *Immunity*.

[41] Nowak A. K., Lake R. A., Marzo A. L. (2003). Induction of tumor cell apoptosis in vivo increases tumor antigen cross-presentation, cross-priming rather than cross-tolerizing host tumor-specific CD8 T cells. *The Journal of Immunology*.

[42] Suzuki E., Kapoor V., Jassar A. S., Kaiser L. R., Albelda S. M. (2005). Gemcitabine selectively eliminates splenic Gr-1^+^/CD11b^+^ myeloid suppressor cells in tumor-bearing animals and enhances antitumor immune activity. *Clinical Cancer Research*.

[43] Ghansah T., Vohra N., Kinney K. (2013). Dendritic cell immunotherapy combined with gemcitabine chemotherapy enhances survival in a murine model of pancreatic carcinoma. *Cancer Immunology, Immunotherapy*.

[44] Nowak A. K., Robinson B. W. S., Lake R. A. (2003). Synergy between chemotherapy and immunotherapy in the treatment of established murine solid tumors. *Cancer Research*.

[45] Fridlender Z. G., Sun J., Singhal S. (2010). Chemotherapy delivered after viral immunogene therapy augments antitumor efficacy via multiple immune-mediated mechanisms. *Molecular Therapy*.

[46] Eric L., Yeo C. J., Lillemoe K. D. (2011). A lethally irradiated allogeneic granulocyte-macrophage colony stimulating factor-secreting tumor vaccine for pancreatic adenocarcinoma: A phase II trial of safety, efficacy, and immune activation. *Annals of Surgery*.

[47] Chen G., Emens L. A. (2013). Chemoimmunotherapy: Reengineering tumor immunity. *Cancer Immunology, Immunotherapy*.

[48] Robert C., Thomas L., Bondarenko I. (2011). Ipilimumab plus dacarbazine for previously untreated metastatic melanoma. *The New England Journal of Medicine*.

[49] Hanna G. G., Coyle V. M., Prise K. M. (2015). Immune modulation in advanced radiotherapies: Targeting out-of-field effects. *Cancer Letters*.

[50] Frey B., Rubner Y., Wunderlich R. (2012). Induction of abscopal anti-tumor immunity and immunogenic tumor cell death by ionizing irradiation - Implications for cancer therapies. *Current Medicinal Chemistry*.

[51] Demaria S., Ng B., Devitt M. L. (2004). Ionizing radiation inhibition of distant untreated tumors (abscopal effect) is immune mediated. *International Journal of Radiation Oncology • Biology • Physics*.

[52] Reits E. A., Hodge J. W., Herberts C. A. (2006). Radiation modulates the peptide repertoire, enhances MHC class I expression, and induces successful antitumor immunotherapy. *The Journal of Experimental Medicine*.

[53] Wennerberg E., Lhuillier C., Vanpouille-Box C. (2017). Barriers to Radiation-Induced In Situ Tumor Vaccination. *Frontiers in Immunology*.

[54] Frey B., Rückert M., Deloch L. (2017). Immunomodulation by ionizing radiation—impact for design of radio-immunotherapies and for treatment of inflammatory diseases. *Immunological Reviews*.

[55] Demaria S., Coleman C. N., Formenti S. C. (2016). Radiotherapy: Changing the Game in Immunotherapy. *Trends in Cancer*.

[56] Van Limbergen E. J., De Ruysscher D. K., Pimentel V. O. (2017). Combining radiotherapy with immunotherapy: The past, the present and the future. *British Journal of Radiology*.

[57] Weichselbaum R. R., Liang H., Deng L., Fu Y.-X. (2017). Radiotherapy and immunotherapy: A beneficial liaison?. *Nature Reviews Clinical Oncology*.

[58] Chajon E., Castelli J., Marsiglia H., De Crevoisier R. (2017). The synergistic effect of radiotherapy and immunotherapy: A promising but not simple partnership. *Critical Review in Oncology/Hematology*.

[59] Deng L., Liang H., Burnette B. (2014). Irradiation and anti–PD-L1 treatment synergistically promote antitumor immunity in mice. *The Journal of Clinical Investigation*.

[60] Demaria S., Kawashima N., Yang A. M. (2005). Immune-mediated inhibition of metastases after treatment with local radiation and CTLA-4 blockade in a mouse model of breast cancer. *Clinical Cancer Research*.

[61] Zeng J., See A. P., Phallen J. (2013). Anti-PD-1 blockade and stereotactic radiation produce long-term survival in mice with intracranial gliomas. *International Journal of Radiation Oncology • Biology • Physics*.

[62] Hiniker S. M., Chen D. S., Reddy S. (2012). A Systemic complete response of metastatic melanoma to local radiation and immunotherapy. *Translational Oncology*.

[63] Postow M. A., Callahan M. K., Barker C. A. (2012). Immunologic correlates of the abscopal effect in a patient with melanoma. *The New England Journal of Medicine*.

[64] Golden E. B., Demaria S., Schiff P. B., Chachoua A., Formenti S. C. (2013). An abscopal response to radiation and ipilimumab in a patient with metastatic non-small cell lung cancer. *Cancer Immunology Research*.

[65] Slovin S. F., Higano C. S., Hamid O. (2013). Ipilimumab alone or in combination with radiotherapy in metastatic castration-resistant prostate cancer: results from an open-label, multicenter phase I/II study. *Annals of Oncology*.

[66] Ling D. C., Bakkenist C. J., Ferris R. L., Clump D. A. (2018). Role of Immunotherapy in Head and Neck Cancer. *Seminars in Radiation Oncology*.

[67] West A. C., Johnstone R. W. (2014). New and emerging HDAC inhibitors for cancer treatment. *The Journal of Clinical Investigation*.

[68] Crea F., Fornaro L., Bocci G. (2012). EZH2 inhibition: Targeting the crossroad of tumor invasion and angiogenesis. *Cancer and Metastasis Reviews*.

[69] He Y., Korboukh I., Jin J., Huang J. (2012). Targeting protein lysine methylation and demethylation in cancers. *Acta Biochimica et Biophysica Sinica*.

[70] West A. C., Mattarollo S. R., Shortt J. (2013). An intact immune system is required for the anticancer activities of histone deacetylase inhibitors. *Cancer Research*.

[71] Magner W. J., Kazim A. L., Stewart C. (2000). Activation of MHC Class I, II, and CD40 Gene Expression by Histone Deacetylase Inhibitors. *The Journal of Immunology*.

[72] Armeanu S., Bitzer M., Lauer U. M. (2005). Natural killer cell-mediated lysis of hepatoma cells via specific induction of NKG2D ligands by the histone deacetylase inhibitor sodium valproate. *Cancer Research*.

[73] Fonsatti E., Nicolay H. J. M., Sigalotti L. (2007). Functional up-regulation of human leukocyte antigen class I antigens expression by 5-aza-2′-deoxycytidine in cutaneous melanoma: Immunotherapeutic implications. *Clinical Cancer Research*.

[74] Coral S., Sigalotti L., Altomonte M. (2002). 5-aza-2′-deoxycytidine-induced expression of functional cancer testis antigens in human renal cell carcinoma: Immunotherapeutic implications. *Clinical Cancer Research*.

[75] Dunn J., Rao S. (2017). Epigenetics and immunotherapy: The current state of play. *Molecular Immunology*.

[76] Filippakopoulos P., Qi J., Picaud S. (2010). Selective inhibition of BET bromodomains. *Nature*.

[77] Zhu H., Bengsch F., Svoronos N. (2016). BET Bromodomain Inhibition Promotes Anti-tumor Immunity by Suppressing PD-L1 Expression. *Cell Reports*.

[78] Hongwei Wang F. C., Cheng J., Alejandro V. (2015). JQ1, a Selective Bromodomain Inhibitor, Augment the Immunogenicity of Mantle Cell Lymphoma By Influencing the Expression of PD-L1. *Blood*.

[79] Kagoya Y., Nakatsugawa M., Yamashita Y. (2016). BET bromodomain inhibition enhances T cell persistence and function in adoptive immunotherapy models. *The Journal of Clinical Investigation*.

[80] Dennis O. A., Gordon J. F., Wong K. K. (2016). BET bromodomain inhibition synergizes with immune checkpoint blockade to facilitate anti-tumor response in a murine model of non-small cell lung cancer harboring activating KRAS mutation. *The Journal of Immunology*.

[81] Lindau D., Gielen P., Kroesen M., Wesseling P., Adema G. J. (2013). The immunosuppressive tumour network: myeloid-derived suppressor cells, regulatory T cells and natural killer T cells. *The Journal of Immunology*.

[82] Noy R., Pollard J. W. (2014). Tumor-associated macrophages: from mechanisms to therapy. *Immunity*.

[83] Mantovani A., Sozzani S., Locati M., Allavena P., Sica A. (2002). Macrophage polarization: tumor-associated macrophages as a paradigm for polarized M2 mononuclear phagocytes. *Trends in Immunology*.

[84] Cannarile M. A., Ries C. H., Hoves S., Rüttinger D. (2014). Targeting tumor-associated macrophages in cancer therapy and understanding their complexity. *OncoImmunology*.

[85] Mantovani A., Sica A., Sozzani S., Allavena P., Vecchi A., Locati M. (2004). The chemokine system in diverse forms of macrophage activation and polarization. *Trends in Immunology*.

[86] Loberg R. D., Ying C., Craig M. (2007). Targeting CCL2 with systemic delivery of neutralizing antibodies induces prostate cancer tumor regression in vivo. *Cancer Research*.

[87] Fridlender Z. G., Kapoor V., Buchlis G. (2011). Monocyte chemoattractant protein-1 blockade inhibits lung cancer tumor growth by altering macrophage phenotype and activating CD8+ cells. *American Journal of Respiratory Cell and Molecular Biology*.

[88] Mantovani A., Marchesi F., Malesci A., Laghi L., Allavena P. (2017). Tumour-associated macrophages as treatment targets in oncology. *Nature Reviews Clinical Oncology*.

[89] Brana I., Calles A., LoRusso P. M. (2015). Carlumab, an anti-C-C chemokine ligand 2 monoclonal antibody, in combination with four chemotherapy regimens for the treatment of patients with solid tumors: an open-label, multicenter phase 1b study. *Targeted Oncology*.

[90] Nywening T. M., Wang-Gillam A., Sanford D. E. (2016). Targeting tumour-associated macrophages with CCR2 inhibition in combination with FOLFIRINOX in patients with borderline resectable and locally advanced pancreatic cancer: a single-centre, open-label, dose-finding, non-randomised, phase 1b trial. *The Lancet Oncology*.

[91] Wang Y., Zhang X., Yang L., Xue J., Hu G. (2018). Blockade of CCL2 enhances immunotherapeutic effect of anti-PD1 in lung cancer. *Journal of Bone Oncology*.

[92] Ruffell B., Coussens L. M. (2015). Macrophages and therapeutic resistance in cancer. *Cancer Cell*.

[93] DeNardo D. G., Brennan D. J., Rexhepaj E. (2011). Leukocyte complexity predicts breast cancer survival and functionally regulates response to chemotherapy. *Cancer Discovery*.

[94] Ries C. H., Cannarile M. A., Hoves S. (2014). Targeting tumor-associated macrophages with anti-CSF-1R antibody reveals a strategy for cancer therapy. *Cancer Cell*.

[95] Stafford J. H., Hirai T., Deng L. (2016). Colony stimulating factor 1 receptor inhibition delays recurrence of glioblastoma after radiation by altering myeloid cell recruitment and polarization. *Neuro-Oncology*.

[96] Pyonteck S. M., Akkari L., Schuhmacher A. J. (2013). CSF-1R inhibition alters macrophage polarization and blocks glioma progression. *Nature Medicine*.

[97] Germano G., Frapolli R., Simone M. (2010). Antitumor and anti-inflammatory effects of trabectedin on human myxoid liposarcoma cells. *Cancer Research*.

[98] Germano G., Frapolli R., Belgiovine C. (2013). Role of macrophage targeting in the antitumor activity of trabectedin. *Cancer Cell*.

[99] Pollard J. W. (2004). Tumour-educated macrophages promote tumour progression and metastasis. *Nature Reviews Cancer*.

[100] Martinez F. O., Gordon S. (2014). The M1 and M2 paradigm of macrophage activation: time for reassessment. *F1000Prime Reports*.

[101] Joshi S., Singh A. R., Zulcic M., Durden D. L. (2014). A macrophage-dominant PI3K isoform controls hypoxia-induced HIF1*α* and HIF2*α* stability and tumor growth, angiogenesis, and metastasis. *Molecular Cancer Research*.

[102] Joshi S., Singh A. R., Zulcic M. (2014). Rac2 controls tumor growth, metastasis and M1-M2 macrophage differentiation in vivo. *PLoS ONE*.

[103] Kaneda M. M., Messer K. S., Ralainirina N. (2016). PI3K*γ* is a molecular switch that controls immune suppression. *Nature*.

[104] Pello O. M., De Pizzol M., Mirolo M. (2012). Role of c-MYC in alternative activation of human macrophages and tumor-associated macrophage biology. *Blood*.

[105] Gordon S., Martinez F. O. (2010). Alternative activation of macrophages: mechanism and functions. *Immunity*.

[106] De Henau O., Rausch M., Winkler D. (2016). Overcoming resistance to checkpoint blockade therapy by targeting PI3K*γ* in myeloid cells. *Nature*.

[107] Tolcher A. W., Hong D. S., Sullivan R. J. (2016). IPI-549-01-A phase 1/1b first in human study of IPI-549, a PI3K-*γ* inhibitor, as monotherapy and in combination with pembrolizumab in subjects with advanced solid tumors.. *Journal of Clinical Oncology*.

[108] Gunderson A. J., Kaneda M. M., Tsujikawa T. (2016). Bruton tyrosine kinase–Dependent immune cell cross-talk drives pancreas cancer. *Cancer Discovery*.

[109] Beatty G. L., Torigian D. A., Gabriela Chiorean E. (2013). A phase I study of an agonist CD40 monoclonal antibody (CP-870,893) in combination with gemcitabine in patients with advanced pancreatic ductal adenocarcinoma. *Clinical Cancer Research*.

[110] Bald T., Landsberg J., Lopez-Ramos D. (2014). Immune cell-poor melanomas benefit from PD-1 blockade after targeted type I IFN activation. *Cancer Discovery*.

[111] Shirota Y., Shirota H., Klinman D. M. (2012). Intratumoral injection of CpG oligonucleotides induces the differentiation and reduces the immunosuppressive activity of myeloid-derived suppressor cells. *The Journal of Immunology*.

[112] Houot R., Levy R. (2009). T-cell modulation combined with intratumoral CpG cures lymphoma in a mouse model without the need for chemotherapy. *Blood*.

[113] Sato-Kaneko F., Yao S., Ahmadi A. (2017). Combination immunotherapy with TLR agonists and checkpoint inhibitors suppresses head and neck cancer. *JCI Insight*.

[114] Gabrilovich D. I., Nagaraj S. (2009). Myeloid-derived suppressor cells as regulators of the immune system. *Nature Reviews Immunology*.

[115] Ostrand-Rosenberg S. (2010). Myeloid-derived suppressor cells: more mechanisms for inhibiting antitumor immunity. *Cancer Immunology, Immunotherapy*.

[116] Nefedova Y., Fishman M., Sherman S., Wang X., Beg A. A., Gabrilovich D. I. (2007). Mechanism of all-trans retinoic acid effect on tumor-associated myeloid-derived suppressor cells. *Cancer Research*.

[117] Huang M. E., Ye Y. C., Chen S. R. (1988). Use of all-trans retinoic acid in the treatment of acute promyelocytic leukemia. *Blood*.

[118] Mirza N., Fishman M., Fricke I. (2006). *All-trans*-retinoic acid improves differentiation of myeloid cells and immune response in cancer patients. *Cancer Research*.

[119] Iclozan C., Antonia S., Chiappori A., Chen D.-T., Gabrilovich D. (2013). Therapeutic regulation of myeloid-derived suppressor cells and immune response to cancer vaccine in patients with extensive stage small cell lung cancer. *Cancer Immunology, Immunotherapy*.

[120] Walsh J. E., Clark A., Day T. A., Gillespie M. B., Young M. R. (2010). Use of *α*,25-Dihydroxyvitamin D3 treatment to stimulate immune infiltration into head and neck squamous cell carcinoma. *Human Immunology*.

[121] Veikkola T., Karkkainen M., Claesson-Welsh L., Alitalo K. (2000). Regulation of angiogenesis via vascular endothelial growth factor receptors. *Cancer Research*.

[122] Gabrilovich D. I., Chen H. L., Girgis K. R. (1996). Production of vascular endothelial growth factor by human tumors inhibits the functional maturation of dendritic cells. *Nature Medicine*.

[123] Osada T., Chong G., Tansik R. (2008). The effect of anti-VEGF therapy on immature myeloid cell and dendritic cells in cancer patients. *Cancer Immunology, Immunotherapy*.

[124] Highfill S. L., Cui Y., Giles A. J. (2014). Disruption of CXCR2-mediated MDSC tumor trafficking enhances anti-PD1 efficacy. *Science Translational Medicine*.

[125] Jung H., Bischof A., Ebsworth K. (2015). Abstract A90: Combination therapy of chemokine receptor inhibition plus PDL-1 blockade potentiates anti-tumor effects in a murine model of breast cancer. *Molecular Cancer Therapeutics*.

[126] Serafini P., Meckel K., Kelso M. (2006). Phosphodiesterase-5 inhibition augments endogenous antitumor immunity by reducing myeloid-derived suppressor cell function. *The Journal of Experimental Medicine*.

[127] Meyera C., Sevko A., Ramacher M. (2011). Chronic inflammation promotes myeloid-derived suppressor cell activation blocking antitumor immunity in transgenic mouse melanoma model. *Proceedings of the National Acadamy of Sciences of the United States of America*.

[128] Califano J. A., Khan Z., Noonan K. A. (2015). Tadalafil augments tumor specific immunity in patients with head and neck squamous cell carcinoma. *Clinical Cancer Research*.

[129] Hassel J. C., Jiang H., Bender C. (2017). Tadalafil has biologic activity in human melanoma. Results of a pilot trial with Tadalafil in patients with metastatic Melanoma (TaMe). *OncoImmunology*.

[130] Schmid M. C., Avraamides C. J., Dippold H. C. (2011). Receptor tyrosine kinases and TLR/IL1Rs unexpectedly activate myeloid cell PI3K*γ*, A single convergent point promoting tumor inflammation and progression. *Cancer Cell*.

[131] Davis R. J., Moore E. C., Clavijo P. E. (2017). Anti-PD-L1 Efficacy Can Be Enhanced by Inhibition of Myeloid-Derived Suppressor Cells with a Selective Inhibitor of PI3K*δ*/*γ*. *Cancer Research*.

[132] Sugiyama D., Nishikawa H., Maeda Y. (2013). Anti-CCR4 mAb selectively depletes effector-type FoxP3^+^CD4^+^ regulatory T cells, evoking antitumor immune responses in humans. *Proceedings of the National Acadamy of Sciences of the United States of America*.

[133] Prendergast G. C., Smith C., Thomas S. (2014). Indoleamine 2,3-dioxygenase pathways of pathogenic inflammation and immune escape in cancer. *Cancer Immunology, Immunotherapy*.

[134] Yoshida R., Imanishi J., Oku T., Kishida T., Hayaishi O. (1981). Induction of pulmonary indoleamine 2,3-dioxygenase by interferon. *Proceedings of the National Acadamy of Sciences of the United States of America*.

[135] Fallarino F., Grohmann U., Hwang K. W. (2003). Modulation of tryptophan catabolism by regulatory T cells. *Nature Immunology*.

[136] Smith C., Chang M. Y., Parker K. H. (2012). IDO is a nodal pathogenic driver of lung cancer and metastasis development. *Cancer Discovery*.

[137] Frumento G., Rotondo R., Tonetti M., Damonte G., Benatti U., Ferrara G. B. (2002). Tryptophan-derived catabolites are responsible for inhibition of T and natural killer cell proliferation induced by indoleamine 2,3-dioxygenase. *The Journal of Experimental Medicine*.

[138] Holmgaard R. B., Zamarin D., Munn D. H., Wolchok J. D., Allison J. P. (2013). Indoleamine 2,3-dioxygenase is a critical resistance mechanism in antitumor T cell immunotherapy targeting CTLA-4. *The Journal of Experimental Medicine*.

[139] Spranger S., Koblish H. K., Horton B., Scherle P. A., Newton R., Gajewski T. F. (2014). Mechanism of tumor rejection with doublets of CTLA-4, PD-1/PD-L1, or IDO blockade involves restored IL-2 production and proliferation of CD8+ T cells directly within the tumor microenvironment. *Journal for ImmunoTherapy of Cancer*.

[140] Gibney G., Hamid O., Lutzky J. (2015). 511 Updated results from a phase 1/2 study of epacadostat (INCB024360) in combination with ipilimumab in patients with metastatic melanoma. *European Journal of Cancer*.

[141] Merck Ia Incyte and Merck to Advance Clinical Development Program Investigating the Combination of Epacadostat with KEYTRUDA® (pembrolizumab). https://wwwmrknewsroomcom/news-release/oncology-newsroom/incyte-and-merck-advance-clinical-development-program-investigating-c.

[142] Incyte Incyte and Merck Provide Update on Phase 3 Study of Epacadostat in Combination with KEYTRUDA® (pembrolizumab) in Patients with Unresectable or Metastatic Melanoma.

[143] Soliman H. H., Minton S. E., Han H. S. (2016). A Phase I study of indoximod in patients with advanced malignancies. *Oncotarget *.

[144] Lebrun J. J. (2012). The Dual Role of TGFbeta in Human Cancer: From Tumor Suppression to Cancer Metastasis. *Molecular Biology*.

[145] Hanks B. A., Holtzhausen A., Evans K., Heid M., Blobe G. C. (2014). Combinatorial TGF-*β* signaling blockade and anti-CTLA-4 antibody immunotherapy in a murine BRAF. *Journal of Clinical Oncology*.

[146] Herbertz S., Sawyer J. S., Stauber A. J. (2015). Clinical development of galunisertib (Ly2157299 monohydrate), a small molecule inhibitor of transforming growth factor-beta signaling pathway. *Drug Design, Development and Therapy*.

[147] Duan Y.-C., Ma Y.-C., Qin W.-P. (2017). Design and synthesis of tranylcypromine derivatives as novel LSD1/HDACs dual inhibitors for cancer treatment. *European Journal of Medicinal Chemistry*.

[148] Gu L., Zhang H., Liu T. (2016). Discovery of Dual Inhibitors of MDM2 and XIAP for Cancer Treatment. *Cancer Cell*.

[149] Weinstock J., Wu J., Cao P. (2012). Selective dual inhibitors of the cancer-related deubiquitylating proteases USP7 and USP47. *ACS Medicinal Chemistry Letters*.

[150] Zang J., Liang X., Huang Y. (2017). Discovery of Novel Pazopanib-Based HDAC and VEGFR Dual Inhibitors Targeting Cancer Epigenetics and Angiogenesis Simultaneously. *Journal of Medicinal Chemistry*.

[151] He S., Dong G., Wu S. (2018). Small Molecules Simultaneously Inhibiting p53-Murine Double Minute 2 (MDM2) Interaction and Histone Deacetylases (HDACs): Discovery of Novel Multitargeting Antitumor Agents. *Journal of Medicinal Chemistry*.

[152] Erdreich-Epstein A., Singh A. R., Joshi S. (2017). Association of high microvessel alphavbeta3 and low PTEN with poor outcome in stage 3 neuroblastoma: rationale for using first in class dual PI3K/BRD4 inhibitor, SF1126. *Oncotarget*.

[153] Singh A. R., Joshi S., Burgoyne A. M. (2016). Single agent and synergistic activity of the "first-in-class" dual PI3K/BRD4 inhibitor SF1126 with sorafenib in hepatocellular carcinoma. *Molecular Cancer Therapeutics*.

[154] Joshi S., Singh A. R., Durden D. L. (2015). Pan-PI-3 kinase inhibitor SF1126 shows antitumor and antiangiogenic activity in renal cell carcinoma. *Cancer Chemotherapy and Pharmacology*.

[155] Garlich J. R., De P., Dey N. (2008). A vascular targeted pan phosphoinositide 3-kinase inhibitor prodrug, SF1126, with antitumor and antiangiogenic activity. *Cancer Research*.

[156] Ciceri P., Müller S., O'Mahony A. (2014). Dual kinase-bromodomain inhibitors for rationally designed polypharmacology. *Nature Chemical Biology*.

[157] Morales G. A., Garlich J. R., Su J. (2013). Synthesis and cancer stem cell-based activity of substituted 5-morpholino-7H-thieno[3,2-b]pyran-7-ones designed as next generation PI3K inhibitors. *Journal of Medicinal Chemistry*.

[158] Andrews F. H., Singh A. R., Joshi S. (2017). Dual-activity PI3K–BRD4 inhibitor for the orthogonal inhibition of MYC to block tumor growth and metastasis. *Proceedings of the National Acadamy of Sciences of the United States of America*.

[159] Shen G., Jiang M., Pu J. (2018). Dual inhibition of BRD4 and PI3K by SF2523 suppresses human prostate cancer cell growth in vitro and in vivo. *Biochemical and Biophysical Research Communications*.

[160] Zhu H., Mao J.-H., Wang Y. (2017). Dual inhibition of BRD4 and PI3K-AKT by SF2523 suppresses human renal cell carcinoma cell growth. *Oncotarget *.

[161] *S. R. Pharmaceuticals*.

[162] Burgoyne A. M., Vega F. M., Singh A. (2017). *Abstract LB-298: The Novel Triple PI3K-CDK4/6-BRD4 Inhibitor SRX3177 Harnesses Synthetic Lethality Relationships to Orthogonally Disrupt Cancer Cell Signaling*.

